# Supplementary material for: Patterns of Intron Gain and Loss in Fungi
Source: PLoS Biol. 2004 Nov 30;2(12):e422. doi: 10.1371/journal.pbio.0020422 (PMC532390; doi:10.1371/journal.pbio.0020422)
Supplement: Table S1 — Also available at http://genes.mit.edu/NielsenEtAl/. (4.3 MB ZIP). [file pbio.0020422.st001.zip › NielsenEtAl/html/670.html]

AN8562.1.NCU08603.1.MG05656.1.FG00550.1


```
 CLUSTAL W (1.82) Multiple Sequence Alignments - Introns Inserted


Sequence 1: NCU08603.1	1900 aa
Sequence 2: MG05656.1	1799 aa
Sequence 3: FG00550.1	1835 aa
Sequence 4: AN8562.1	1764 aa
Alignment Length: 1979 aa
Number Identitical Residues: 515 aa
Alignment Score (without introns) 28990


MG05656.1 	------------------------------MSSTKLPATPVPHTELPAYLEKHNRAPVVD
NCU08603.1	------------------------------MATSTFPQLPVPHSDLVKHITNHPQTPMVD
FG00550.1 	--------------------------------MANLPELPAPHSQLAKYISDHKDTPITE
AN8562.1  	MMALTSQPWLSINPSSSITALSIPIYNRFMTTLSSLPSLPAKHSQFLQYVASHPNTPMPE
          	  : ::..  : ..::: :: : .  .    : :.:*  *. *:::  :: .*  :*: :

MG05656.1 	LFKPYRVFENELRALYAQEPDNEALKDPYVNVLPLFASH-MPEIKTRARNLKRESAEERS
NCU08603.1	LLQPYRDYEARLRQVYAQEPDSELLKDPYVNVLPLFTDN-TSDIKIRARNMEAESEEERA
FG00550.1 	ILEPYRKYEAELRSVYAQDRQNPILDDPYLNVVPLFTED-TKFITTRARNLEAESEEEKS
AN8562.1  	LVRPYNDYDAQLRKIFAQEPTHPAVADNIVNIVPLFDANGSADVRIRARDLASESETTRS
          	:..**. :: .** ::**:     : *  :*::***  ..   :  ***::  **   ::

MG05656.1 	KYIMALPDDKRRHNGSPALVDSLKQFRHNFAVFSETSLLDLNWDNVVVAGSAVVNCLLPV
NCU08603.1	RYIMPLPKDIRRPDQSPAVVQSHKDFQRNFNIFSESSLVELNWDNVVAAGSSVVNCILPV
FG00550.1 	KYIMALPDEKRRPHDSPATVADLAEFQKNFNIFSESSLADLDWSNVVAAGSSVVNCLLPV
AN8562.1  	KYLMPLKDEHRKPTGVPAVVSHIKNFQTNFNIFSESSLTDLDWNNVVAAGSAVATSLLPV
          	:*:*.* .: *:    ** *    :*: ** :***:** :*:*.***.***:*...:***

MG05656.1 	PHEFNKSKRTLREYYHEKLCPASDVDLFLYGLTEEEAIEKIKEIEASVKDSLLTECTTVR
NCU08603.1	PKKYSGSKRLMREFYHEKFAPASDVDLFLYGLTEAQAIEKIKEIESQVRDALLCETTTVR
FG00550.1 	PKEFNTTKRKLREYYHEKFCPASDVDLFLYGLNHDQAIEKIKKIERAVKDALLNEVTVVR
AN8562.1  	PDKYSDSKRGMRQFYHEEFAPASDVDLFLYGLTEDQAIEKIKQIERCIRDSILTEVSTIR
          	*.::. :** :*::***::.************.. :******:**  ::*::* * :.:*

MG05656.1 	TKHAITICSQYPTRHIQ~IVLRIYKSVSEILTGFDIDSSGAAYDGK-~-QVYCTPRALQS
NCU08603.1	TKHAVTICSQYPTRHIQ~IVLRIYKSVSEILTGFDIDCSGAAYDGK-~-QVYCTPRALQS
FG00550.1 	TKYAITIASQYPVRHIQ0IVLRVYKSVSEILTGFDIDAAGGAYNGSQ0CQVYVTPRALGS
AN8562.1  	TKNAITIVSQHPTRHVQ~IVLRRYKSIAEILTGFDVDCSCAAYDGS-~-QVYLAPRAVGA
          	** *:** **:*.**:* **** ***::*******:*.: .**:*.   *** :***: :

MG05656.1 	YMTQINHIDLSRRSPSYENRLSKYSHRNFEVYWPDLDRSRVDP~TIFERNFRRTLGLARL
NCU08603.1	YMTQTNHIDLTRRSPSYENRLSKYSRRGFEVYWPELDRSRIDP~TIFERSFMRTLGLARL
FG00550.1 	FITQINHIDLTRRSPSYENRLSKYSKRAFEIYYPDFSREKVDP~TIFERSFRRTLGLARL
AN8562.1  	FITQVNQIDLTRRSPSYENRLSKYSHRGFEIFWPDLDRSRIDP0SIFERSFTRTVGLARL
          	::** *:***:**************:* **:::*::.*.::** :****.* **:*****

MG05656.1 	LVLERLPTSSARDDYLAKRRVERGRPPIDRDFWS--GGLRGNIKDRHEDEVADWVNEEEM
NCU08603.1	LVLERLPTAKARDNYVDQRRQERGRPALNRFVRKNFRAMGSNIKDDYDDEIADWVSGDEV
FG00550.1 	LVLERLPTTTARETYLDKRREERGRPRVYRQRQV----LHGNIKDYHEDEVADWLSEEDV
AN8562.1  	LVLEKLPKSSDREEYLAQRRKERGRPPPNTFYRP--NRLAGNIKNDWQDEIAEWVDEDEV
          	****:**.:. *: *: :** *****            : .***:  :**:*:*:. :::

MG05656.1 	SNYHTF~TIPYGVKFHAKKIEKLCYTRDLL1LNAEWNQPKEREVYLHRHPAF~FGRVIDI
NCU08603.1	SDYHTF~TIPYGPKFHAKKIEKLCYTKDLL~LNAEWNQHDNRTVYLHRHPAF0FGRFKDV
FG00550.1 	SNYHTF~TVPYGQHFNAKRIEKLCYTRDLL~LNAEWNQPDERKVYLHRHPAF~FGRVEDV
AN8562.1  	SDYHTF0TIPYGPKYHARKIEKLLYTKDLL~LNAEWNKPKDREVNLHRHPAF~FGRAEDV
          	*:**** *:*** :::*::**** **:*** ******: .:* * ******* ***  *:

MG05656.1 	IEDCCGFCPTPHTEEELAVHEEESKVYVSGRISFIQDDPGRQQIGSFNPLSDDNWTE~MA
NCU08603.1	VEDCCGHCPVPQTDEEKDIAEEEEKIYVSGRISFIKDDPGRQQIGSFNPLTDDDWTE~MA
FG00550.1 	IEDCCGCCPEPVTDDEKEVAEKEGEIYIKGKVSFLIDDPGRQQIGSFNPLTENDWTD~MA
AN8562.1  	MHDCCGFCPKPTTSEEEEVAAEESKIYISGDVSFLSDNPGRQEIGSFNPITDTDWTE1MA
          	:.**** ** * *.:*  :  :* ::*:.* :**: *:****:******::: :**: **

MG05656.1 	YVGNTARLCQAIVDLDAEHVEDWIAQEGADVNQRDYTGRTPLHLAVTSSTPEIVALLIKA
NCU08603.1	YIGNTARLCQAIVDEDVEHVQDWLAQEGANPNTRDHTGRTPLHLAVMSSSPAVVRALVEA
FG00550.1 	YVGNVTRLCQSIVDGDVEDVSNWLSQEDSDPNKRDYTGRTPLHLAVMASTPEVVKCLVDH
AN8562.1  	YVGNTAGLCQAIVDNDLEEVQRWLAQGEIDVNRRDHTGRTPLHLACMTSTPEIVQHLVDR
          	*:**.: ***:*** * *.*. *::*   : * **:*********  :*:* :*  *:. 

MG05656.1 	GARLIARLADGRTALHLAAVRGDLEIVKMLLDKSNENEDEEEMKRDLRRKQTAPALSEQT
NCU08603.1	GARLTARLADGRTALHLAASRGDVEIVKILMNKSNANEEEFEELEDQRRKARAALREKKT
FG00550.1 	GARLTARLADGKTALHLAASRGDSSMIKILMEKSIENEEAEEERQDKKRQAAKKAVASNK
AN8562.1  	GARLIARLADGRTALHLAAARGSVEIVRILLTKSEQNEEEESRKENAQKAARKVKKDDAS
          	**** ******:******* **. .::::*: **  **:  .  .: ::        . .

MG05656.1 	EQHSAPEEKHDEEAEDSDNESAEILSDEGSDEDDEAHSMATGSFVKIKRKGGDSYNQDLI
NCU08603.1	EKMDIDEEKADDSEDIEMIEAGDAESDAGGDK----MSIATGSFVKVDKTPETPAPDGIL
FG00550.1 	DGDSEDPAGSEANEDSD----GELIDGETTDVDS--ASMATGSFVKVKEAG-DNNDEDLV
AN8562.1  	ENSDE-----------------EMFDVSSDEEDSYAHSNTTGSFVKVKKEQ-DDEQAVAP
          	:  .                  :  .    : .. : * :******:..           

MG05656.1 	PEEKKDDPDIYDVNVIAWDTPCSALHFAIVSGNDEVVKLLCQQ~YGADVLLPVKYFG-HD
NCU08603.1	VAEDEEGPDFYKVDEVAWDIPCSALHLAIMEGHCEVVKLLCQE~YGADVLLPVKFPQ-DK
FG00550.1 	PDESQDEPDFFDIDALAWDLQCSPLHLAIAEGNEDAVRTLC-D0FGSDSIMPVKSLT-DG
AN8562.1  	DDADELDPDIYDINVLAWDNKASPLHLAILNGHVAVVDELVTS~FGADVLLPVKLVHSYS
          	   .:  **::.:: :***  .*.**:** .*:  .*  *  . :*:* ::***   :  

MG05656.1 	NRPTAALLTLVMALKLPGEKAKSMVKTLLSLGATSAQADMNGFTCFHQYAEAGATELIDL
NCU08603.1	NK---VLLTLGLVMALPIEKAKSMAQTLLSLGATVAQADMNSCTVFTRYVERNARTLLDL
FG00550.1 	DAQ--AILTLVLALSLPSDKAKSMANLLLELGATSAQADSKGCSVFHRYVEAGEEDLVDT
AN8562.1  	NSPRAAILTLVLAMRLPLDAAKAMTEKLLQVGASPAQADINQKTALHYLAHYGRHELLDI
          	:   :.:*** :.: ** : **:*.: **.:**: **** :  : :   .. .   *:* 

MG05656.1 	LKELDRTGVNNSINHVAVF---RYGKSFGPLQSAIKNGDTGLVVQLLEAGAAAQIDFETW
NCU08603.1	ACELDKAGYKTAINHVCN----QWAQSETPLLIAIKDGDLSMVVKLLETGAVPEIDFETW
FG00550.1 	LLANDKTGIKAAINHMVCGGSYWNSEAIAPISTAIENGDVALVLKLLTAGAKSEIDFDTW
AN8562.1  	YKQYDQPAVDRAINHLSTTGSYYSFSAVSPLTHAITAKDRTKVMKLLEMGAHPAISLEDY
          	    *:.. . :***:   .:    .:  *:  **   *   *::**  ** . *.:: :

MG05656.1 	LNGAK-QSSEYDIVSASES-EKFFRRSTEQPIILAALYGHPFIIMELLNHGADVNTISAQ
NCU08603.1	LKSAR-QSPKESRLSDFESNKKLFQEDLDRPLIAALRSKEPSIALELLERGCDPNPLHPE
FG00550.1 	LKSAK-VSGMSGRLGDLERNQQMYSEGAEQPLITAIRLGNTEIAIKLLENGADPNPLDIP
AN8562.1  	LKAAKSLEYSLGNHPSVVDHKREFELNILQPVILAIELDMPLVAAELLARGVDPNTLTRD
          	*:.*:: .   .        :: :  .  :*:* *     . :  :** .* * *.:   

MG05656.1 	SQDLLDPNSYSRGYNSGQTLLDILRKQLETLRLYKGEEPRISEPLPFTGDD-KALEGAKP
NCU08603.1	SVRGLHS-TYSYRWK-GKSALDMVREQLEDLRKYKGENTKFEEPKPRLGLD-TYLQRFEE
FG00550.1 	THELIFN-EYRRDYNKGRSALDLVRSSLKKLRGYKGEKRQPVEPKETPGLD-EFLSRFTE
AN8562.1  	GYRVLRN-EWSRNDTSGSSLLDYVQAKVTALRKYKGEKVCAQKPIELDPDDSTYLQGLQE
          	    :    :    ..* : ** :: .:  ** ****:    :*      *:  *.    

MG05656.1 	GSWKHLVVTNDIEAARQEHAERVNIYKALVRQLDALEGLREKKAAILDLIHAFEQAESTI
NCU08603.1	GTYQHWAVSGGVKVEKMGDPEKRTEYEIGLKCLERTKGIPEKEQAVRDAIATLERIEQSI
FG00550.1 	GTYSHCLVSCEVEGERYNFVEGKKKYEKEIKKKREPKGVSEKREAIRELVAGFEAVEEAL
AN8562.1  	DTYKMWLARESLRQKRERFAKEQKIYEDDVKEKENGEGIAEKKAAIEALLADYEKLETTL
          	.::.   .   :.  :    :  . *:  ::     :*: **. *:   :   *  * ::

MG05656.1 	VEKGGKTFSELHPDIP-----DGTRRTNNYWNRANYNSDV----PYEFNHGFTHVKDMTP
NCU08603.1	ISKGGKTFKELYPDRKEDGNDDTSINHHGFWLGRHHDDEVSKKEPYHYNFVFTGVQDMTE
FG00550.1 	ISRGAKPFTELRPDIKTNGREDSSSRNS----RRTDDKDP---KPFEYRFSFNNDRDMTE
AN8562.1  	LSKGAKTFKQLHPDIDYPLNNPRQSRPY-----RSPQLEP-----YKTHFGFH-VELCTD
          	:.:*.*.*.:* **      .    .          : :      :. .. *   .  * 

MG05656.1 	AREAAYVQL~FEAAWDGDLETIKRFTLGAWDADRREAPLMISVKDKNYSNVFGLAFLRGH
NCU08603.1	TKQAAYMEL~FEAVWTGDLDKVKKFTLTSWDDAKQQPPLKIAVEGHNGHNPFTLALQCGH
FG00550.1 	RRRDGYIEL2MEAAWAGDLDKIKQLSLQAWGPEQHQAPLKIAVSDNKGNTPFSIAVLRGH
AN8562.1  	EIKEGYLQL2FEAAWVGDIETIKSLTLSNWGPENQQPPLEIGVTDLSNYSAFTLAVIRGH
          	  . .*::* :**.* **::.:* ::*  *.  .::.** *.* . .  . * :*.  **

MG05656.1 	LRIAKAILEIAQAQYTPEEGTT-TKFTMRDPGYESEDSELPEGDTD-----GPRIYKLIV
NCU08603.1	YELAKAVLEIAQAQYSPPEKAK-TRYSMNKSGGEEEEDDDDDSSSDES---APRIVGETV
FG00550.1 	YDVAKALLEIVKAQWSPPKKDK-VRFRMDTGDDDDEEYSEDSDDSDGSQNGEPRIVSEKV
AN8562.1  	YEAAKAIIAISIAQFKPGEQQKNTRYRIADIDEDSDADSED-----------IELETHVV
          	   ***:: *  **:.* :  ...:: :   . :.:  .              .:    *

MG05656.1 	DEKFTIDNIGEISMQVKSRTKPLDMLVEGSSPFTIINDEGK-------------EQRNTP
NCU08603.1	DDQLTIDTIGQVSMQVESHTLPVSLLRGRTATNGLNYGRWRNHMQLNKGQVLDDEEETWD
FG00550.1 	QQQFTIDNIGHVDLNVESHTKPLQVICQLVPTQWSVKNERLFY-----------HWINTS
AN8562.1  	DDQFTIEDIGEKKNQVESKISPETILQGTCSLKLFLGGIQELD------------NRRIS
          	::::**: **. . :*:*:  *  ::    .      .                      

MG05656.1 	LLFYVIAQNDLNGLTFLLDTATQLSGQRLD----HETETTRFFSCPGQTLNWTIRNGRTE
NCU08603.1	PLMYAVTNNDLKLLKFVLDMGEYFGTLQLD----PATEPPKFFSAPDEVFATAIRKGRVE
FG00550.1 	LITHMLVKDNMTGLKFLLDLAQHYSSQKFEGDDGEDDDSPTGFVFPQDGFKYAVDHGKTK
AN8562.1  	LLEYAIWKDDIELLKFLITLGQELSARKND------KHHSEIYSISHHDFNLAMRLGRVR
          	 : : : ::::  *.*::  .   .  : :       . .  :  . . :  ::  *:..

MG05656.1 	LLAEIIKTTGAGLPLEDLVKRSGAKMIEKPKSYQGLTVYGKKR~KDWATAGRDVMIKARE
NCU08603.1	VMAELIRRTGAGLPLEHLVEGSGVELKEQPRYYQGLSVYGKKR~KDWANAGREVAQQPRD
FG00550.1 	MLALIIKQTGAGIPLDHLVKKSGVELNRKPRYYQGLTVYGRKR2KDWATAGRGMVTRSSG
AN8562.1  	LLEEMIKRTGAGLPIDKLAENSGVEVKEKPKYYQGLSVHGKKR~ADWAAAGGGMPVTRAI
          	::  :*: ****:*::.*.: **.:: .:*: ****:*:*:**  *** **  :      

MG05656.1 	SN--TPPLLTAASCGSIESVEWFLSDTPLRHYLEFANSKGARDDPRLKHLGSTAGGVERA
NCU08603.1	ENSTTSPLLRAAESCCIESIQWFLSDAPLRHYLEFAKSKAAQEDTRMQHLGRAPGGVEGA
FG00550.1 	PR--TPPLLYAALAGNLDSVEFFLGDAPHRLYSEFAKSKASKEDYRFKHLKESPGGFDRA
AN8562.1  	DM--SPPLLLAARKGNLAVVEWFLGTAPGRYYTEFART--HRKDPRLRKLSMSAKGAEQS
          	    :.*** **    :  :::**. :* * * ***.:   :.* *:::*  :. * : :

MG05656.1 	ITKWLGNQN~DLVMHAAIIGGPVGEKTKKLVQYLIKAYPASMTTKSRDGYTPFYLACLRG
NCU08603.1	IARWLDDQG1ELIIHAVIVKSGQ-VDVSHLVSYLIKACPEALEAKSHN-VTPLVQAVYMG
FG00550.1 	ISKWLGADN1DLVIHCAVLANPS-ENANEVLEFLTEAFPAAIEKKNTEGDTPLMVACRLG
AN8562.1  	ILEWLSSRR1DLVLHCAILCDDITDESNALVEYLVKTMPQCLEVRSLDDYTPLGLAFQTH
          	* .**.    :*::*..:: .    . . ::.:* :: * .:  :. :. **:  *    

MG05656.1 	RTDIVELLASH-SDQAVKGPHWENILHAVLKG--CPHADQLQPMLELFEPDLRKAMFKER
NCU08603.1	RVELVKLLLEAGADPNVRTSSWTNILHAALLY--TPPADKLKELLSLFDREAVIRMVTER
FG00550.1 	RIQAVKILLSADADQSARNQKGENILHVALAC--HPKDYQLRELLDLLDTELRSHLFLQK
AN8562.1  	HLTAAKILIAAGADQTVRCKDGMNLIHLALSPKRARKEVTLSDMLSLIDPRLFPSMLTER
          	:   .::*   .:*  .:     *::* .*  .       *  :*.*::      :. ::

MG05656.1 	SSLSAEGRTPLHQWIEKAIGQNWRGSSPQYK------------KPEDLVAVLKMLLEYSG
NCU08603.1	SSLASDGRTPLHQWLT------CIGGNEDHN------------KSVAVFNLLVEMSSEAV
FG00550.1 	KNLAENGATPLHAWVSQASGVQPDNSNSYHRRNYYGYRGAYDGKEQEMIRMLNLILEYSK
AN8562.1  	CSSHQGSLTPLALWMT-------RHHHSDRN-------------AERILQTILDLAEPTG
          	 .    . ***  *:               .                :.  :  : . : 

MG05656.1 	GEELAMLDGTGDTPLHMLISRQAPLETIKLILEMDANLLFRENAVGRTPAELAHDIFIET
NCU08603.1	TTALNMLDASGDTPLHDMLTSHTSSKLIRAVLDFQPSLLFRENAVGRTPVEVVHDELISS
FG00550.1 	GDELEMLNGAGDTCLHTAIMMDM-IALTQALIKFRPSLVYRENAVGRTPAELAHDRLTSQ
AN8562.1  	QKHLELLDGSGNTVIHRAV-NELECGALKEFLSRRPDLLHRENAVGTTPAELAENRWVAK
          	   * :*:.:*:* :*  :  .      : .:.  ..*:.****** **.*:..:     

MG05656.1 	RIGTRN--SGNDRRYYNNDRSEGLDTLINMRPLFFLPDQQAKFKQR-RMQDLNYTSSHAS
NCU08603.1	RVKSESGQQGRYHSRYSVSGAEGYRYLMGEKPQVFLNNMKLYQEKRSREHESQYSDSHNT
FG00550.1 	KFEMPSSPSRSQKDRARDVLRDGPENFIREVEIEAHTPEQRREFLETLGLSDNYKASEAS
AN8562.1  	MTSDPPIAPHPSRYCSYQNIDLSNCSILYKWPREFAKKDKPAGYSKTWTERDAHG-----
          	            :         .   ::           :     .:      :      

MG05656.1 	------------------------------DDQWRQYQGQWAAENR------ERKEDIHQ
NCU08603.1	RMWALCQEYLAKLASTPPPSRSASPPTTEGQTQAHHARDQSPPKRRKLTTETEGRLNAHI
FG00550.1 	-----------------------------LLQISMGIKKQRSIKSLDDKTVGRILWDLCR
AN8562.1  	--------------------------------------------------------ICTE
          	                                                            

MG05656.1 	MCLAYSNGKRRLVSLNEANDVARRLGESFQRGRYYSGMP----KGHDDGAVSEAGQEEN-
NCU08603.1	IATHKLKEKRTLVSLPEANDVAKRLGEMKVGSKYNYQLAPPEEEGQEEEDVKRFADDETP
FG00550.1 	TAMQNHPGKRRLVTLNEANDVAKRLGEKYSASRYFSVQARVE-DGEEDPEENENNTT---
AN8562.1  	IWKQQRGAKRKLVSLFDANEVAKRLAVREGS------------DANRRYRRRRYRHSEN-
          	        ** **:* :**:**:**.                 ...      .    .. 

MG05656.1 	---RPS-------------------QDFVQENYNLICSKWWSKDEKY-------------
NCU08603.1	FASKPDRRTWGDGVRYGRWRKQNGEKQTPRKSYRVLANVAWKDLKKSGEKADDGLDWGRF
FG00550.1 	-------------------------VDFAVQQMNSCLSLAWHENKQPREGKDSSSSDSDE
AN8562.1  	-------------------------YEEVDGELKGDEVMQWL------------------
          	                          :    . .      *                   

MG05656.1 	~----------
NCU08603.1	1YCNLLEQFAS
FG00550.1 	~----------
AN8562.1  	~----------
          	
```
